# The Knowledge of Autism Questionnaire-UK: Development and Initial Psychometric Evaluation

**DOI:** 10.1007/s10803-024-06332-3

**Published:** 2024-05-02

**Authors:** Sophie Langhorne, Nora Uglik-Marucha, Charlotte Broadhurst, Elena Lieven, Amelia Pearson, Silia Vitoratou, Kathy Leadbitter

**Affiliations:** 1 Division of Psychology and Mental Health, University of Manchester, Oxford Road, Manchester, M13 9PL UK; 2 Psychometrics & Measurement Lab, Biostatistics and Health Informatics Department, King’s College London, London, SE5 8AF UK; 3 Department of Psychology, Communication and Human Neuroscience, University of Manchester, Oxford Road, Manchester, M13 9PL UK; 4 Division of Nursing, Midwifery & Social Work, University of Manchester, Oxford Road, Manchester, M13 9PL UK

**Keywords:** Autism, Knowledge, Understanding, Questionnaire, Measurement

## Abstract

Tools to measure autism knowledge are needed to assess levels of understanding within particular groups of people and to evaluate whether awareness-raising campaigns or interventions lead to improvements in understanding. Several such measures are in circulation, but, to our knowledge, there are no psychometrically-validated questionnaires that assess contemporary autism knowledge suitable to the UK context. We aimed to produce a brief measure to assess between-respondent variability and within-respondent change over time. A pool of questionnaire items was developed and refined through a multi-stage iterative process involving autism experts and a lay sample. Attention was paid to face validity, clarity, consensus on correct responses, and appropriate difficulty levels. Initial validation data was obtained from a lay sample of 201 people. Difficulty and discrimination ability were assessed using item response theory and low-performing items were removed. Dimensionality was evaluated with exploratory factor analysis, which revealed a one-factor structure of the questionnaire. Further items were removed where they did not load strongly on their main factor. This process resulted in a final 14-item questionnaire called the Knowledge of Autism Questionnaire-UK. Internal consistency was satisfactory, and the final questionnaire was able to distinguish between parents of autistic people and those without an affiliation to autism. The KAQ-UK is a new, freely-available measure of autism knowledge that could be used to assess between-respondent variability and within-respondent change over time. Further evaluation and validation of its measurement properties are required.

**Supplementary Information:**

The online version contains supplementary material available at 10.1007/s10803-024-06332-3.

Autism is a common and heterogeneous neurodevelopmental condition, affecting around 1.7% of the UK population (Roman-Urrestarazu et al., 2021). Public awareness of autism has somewhat improved over recent decades, with UK polls suggesting that 99.5% of people have heard of autism (NAS, 2015). However, research suggests that only 16% of autistic people in the UK feel understood by the public, resulting in social isolation (APPGA, 2019). Many misconceptions and myths about autism persist, including that autism is a childhood condition, that autism is caused by vaccines, and that autistic people do not enjoy social contact (Autistica, 2019). Accordingly, autistic people are often met with stereotypes and misconceptions (Draaisma, 2009) which may lead to prejudice and stigma. These misunderstandings can also lead to disparities in access to diagnostic and care services (Malik-Soni et al., 2022). In addition to its public health importance, knowledge of autism is needed by autistic people themselves, to aid their self-understanding and self-advocacy. It is also beneficial to the people around the autistic individual, to ensure the individual is understood and well supported.

Several measures of autism knowledge have previously been developed and are in circulation. Previous questionnaires have targeted knowledge in particular groups such as parents and caregivers (e.g., Kuhn & Carter, 2006; Vijayarani, 2013; Wang et al., 2012) or medical professionals (e.g.,Igwe et al., 2010; Shah, 2001), with only a handful of assessments assessing the knowledge of the general adult population (e.g., Gillespie-Lynch et al., 2015; Holt & Christensen, 2013; Mitchell & Locke, 2015). A systematic review by Harrison et al., (2017a, 2017b) found that 57% of assessments of autism knowledge had weak or no psychometric support, with only 7% showing high levels of psychometric support. In addition to a lack of reliability and validity testing, Harrison et al., (2017a, 2017b) reported that measures of autism knowledge were commonly subject to ceiling effects that render questionnaires insensitive to change over time (for instance, the Maternal Autism Knowledge Questionnaire; Kuhn & Carter, 2006).

Following the review by Harrison et al., (2017a, 2017b), research has attempted to address the identified gaps through the development of new measures. More recent measures do not show ceiling effects (e.g., McClain et al., 2019) and have shown sensitivity to change over time (Gillespie-Lynch et al., 2022; Harrison et al., 2019). Extensive psychometric validation has also shown that one measure, The Autism Stigma and Knowledge Questionnaire (ASK-Q; Harrison et al., 2017a, 2017b), has sound psychometric properties, with items holding good discriminatory and difficulty values (see Harrison et al., 2017a, 2017b, for more detail). In addition, a number of measures have good internal consistency in US populations (Benallie et al., 2020; Gillespie-Lynch et al., 2022; Kitchin & Karlin, 2022; McMahon et al., 2020). However, little research has assessed whether internal consistency of these measures withstands in other cultures. Of the research that has taken place, autism knowledge measures developed within the US have been shown to have borderline acceptable internal consistency in other countries (Cage et al., 2019; Kim et al., 2021). It is important to note the ASK-Q (Harrison et al., 2017a, 2017b) has recently been found to hold good internal consistency in Eastern cultures (Harrison et al., 2019; Lu et al., 2020; Su et al., 2023). However further research is needed to further establish the cross-cultural validity of the measure (e.g., Saade et al., 2021).

In addition to limitations around cross-cultural validation, an additional challenge concerns the content of existing autism knowledge questionnaires. Some tap into constructs outside of ‘pure’ autism knowledge by including items that assess awareness of stigmatising beliefs (e.g., ASK-Q; Harrison et al., 2017a, 2017b) or focus specifically on autism symptomatology (e.g., the Revised Autism Symptomatology Knowledge Assessment; McMahon et al., 2020) and therefore may not be appropriate for measuring knowledge more generally. Furthermore, some measures of autism knowledge also include ambiguous items, which are not readily true or false when the diversity of autism presentations and experiences are considered (e.g., “The biggest problem with diagnosing autism is that symptoms do not appear until age 5 or older”; Motta et al., 2018) thus complicating the validity of scoring such questionnaires. Moreover, many questionnaires that have been developed in the US contain country-specific items (e.g., “Less than 2% of people in the US have autism spectrum disorder”, McClain et al., 2019), or include items regarding US practice, such as “Autism can be diagnosed as early as 18 months of age” (Gillespie-Lynch et al., 2022), and “Behaviour therapy is an intervention most likely to be effective for children with autism” (Harrison et al., 2017a, 2017b). Although such items may hold true within US conceptualisation and practice, they may not translate to those of other countries. For instance, autism is very rarely diagnosed as young as 18 months in the UK (Male et al., 2023) and the UK does not readily offer behavioural therapies such as ABA to families (Keenan et al., 2015). As a result, these assessments do not necessarily reflect contextual autism knowledge in countries outside of the US.

A further limitation within current measures of autism knowledge concerns their length. Many research studies use a large battery of assessments when assessing the effectiveness of an intervention or awareness-raising programme and lengthy autism knowledge questionnaires (e.g., ASK-Q, Harrison et al., 2017a, 2017b) can prove be burdensome to administer. Although briefer assessments have been developed, such as the Participatory Autism Knowledge Measure (Gillespie-Lynch et al., 2022) and the 10-item version of the ASK-Q (Love et al., 2020), further psychometric analyses (such as Item Response Theory analysis) are needed to further assess the psychometrics of these measures.

Taken together, the above literature suggests that currently there is no brief, psychometrically validated questionnaire that measures contemporary autism knowledge suitable to the current UK context. A measure of the level of autism knowledge within the UK could help inform policy and practice, as well as allowing researchers to measure whether awareness-raising campaigns or interventions are successful in improving autism knowledge within specific targeted groups or the general public. We therefore set out to produce a valid brief measure of autism knowledge to assess between-respondent variability and within-respondent change over time.

## Aims of the Current Study

The aims of the study are divided into two parts:

### Part 1: Development of Questionnaire Items

To develop a pool of questionnaire items to measure autism knowledge with high face validity (reflecting contemporary UK autism knowledge) by:

- selecting appropriate items from previous relevant questionnaires;
- working with autism experts to generate additional items, assess the appropriateness and clarity of items, and achieve initial consensus on “correct” item responses;
- piloting the item pool with the general public to assess the appropriateness of the difficulty of the questions for intended participants.

### Part 2: Psychometric Analysis

To evaluate the psychometric properties of the measurement tool and its items using contemporary psychometric methods, such as item response theory (IRT) and factor analysis. We aimed:

- To use IRT to select the best performing items, that is, the items that measure the level of autism knowledge evenly and reliably across the continuum of knowledge.
- To identify the number and the nature of the dimensions that represent the construct of autism knowledge, and to retain items that are meaningful indicators of the construct.
- To evaluate the final questionnaire with respect to internal consistency and ability to distinguish between groups based on their affiliation to autism (discriminative validity).

## Methods

Ethical approval for this study was granted by the University of Manchester Research Ethics Committee (Refs: 2019-6116-10034 and 2020-6116-10034). The methods are structured in two parts consistent with the aims above.

### Procedure

#### Part 1: Development of Questionnaire Items

**Fig. 1:**
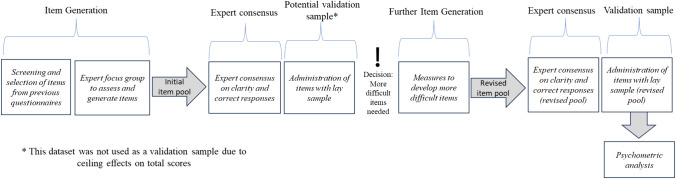
Overview of the process of questionnaire development

**Fig. 2:**
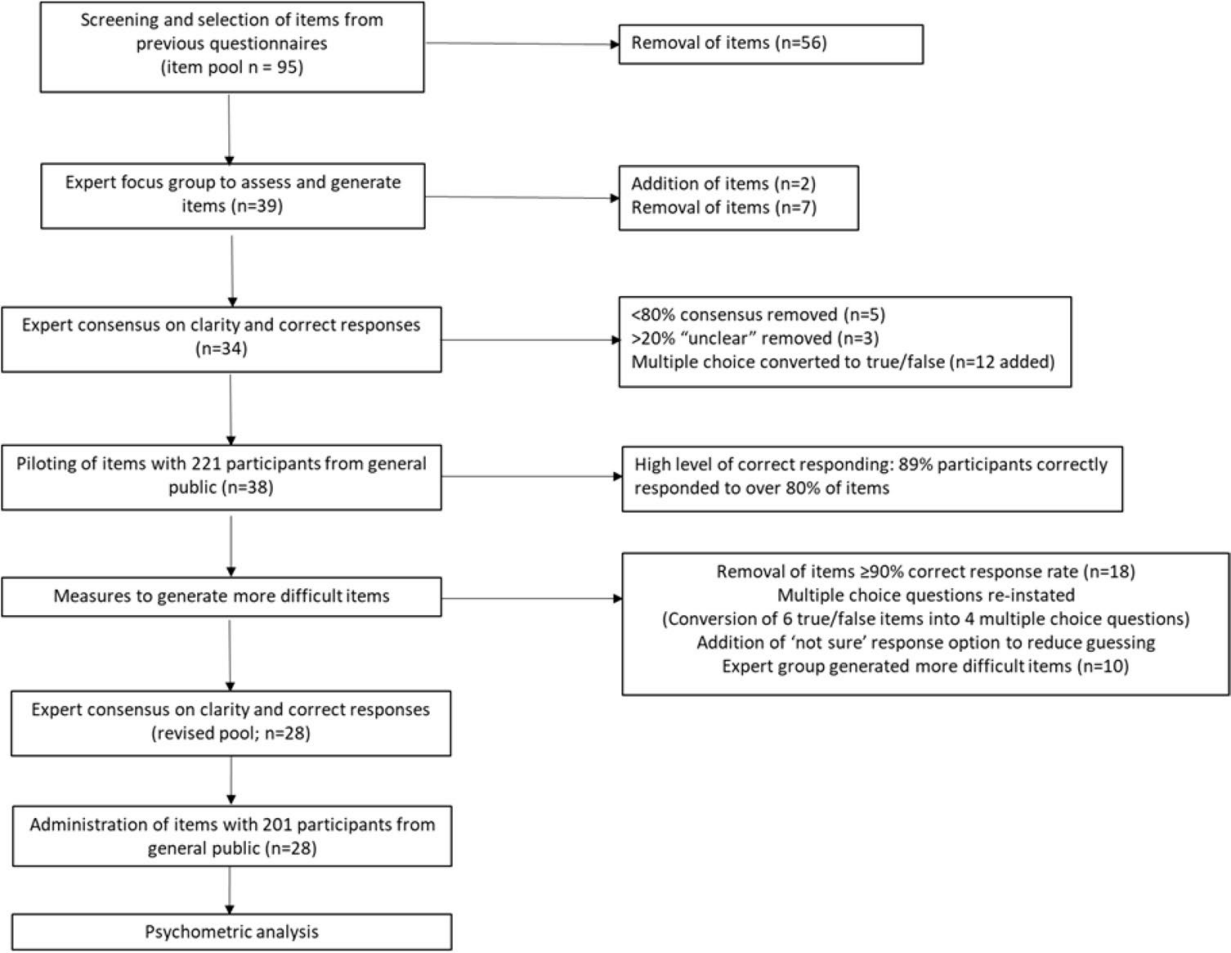
Flowchart of item development (in parentheses, numbers of items in the pool at each stage)

The development of questionnaire items was addressed through a multi-stage process. Figure 1 provides an overview of the stages of this process, with detail provided in the sections below. Figure 2 provides details of item development (addition and removal of potential questionnaire items at each stage).

##### Screening and Selection of Items from Previous Questionnaires

A literature review identified five pre-existing questionnaires that met the following criteria: (1) UK questionnaires published in a peer-reviewed journal from anytime up until October 2018 (the date on which the screening stage took place) or (2) international questionnaires published between 2015 and October 2018, and the questionnaire was available within the paper or upon request (Gillespie-Lynch et al., 2015; Harrison et al., 2017a, 2017b; Helps et al.,1999; Johnson & van Hecke, 2015; Shah, 2001). Items (*n* = 95) were extracted from these five questionnaires and subjected to an initial screening process by two co-authors. Items were removed if they met one or more of the following criteria:

(a) duplicating other items either within or between questionnaires, either verbatim repetition or repetition of content (*n* = 28); for instance, the item ‘Autism is more frequently diagnosed in males than females’ was found in both Gillespie-Lynch et al. (2015) and Harrison et al., (2017a, 2017b).

(b) assessing something other than knowledge (*n* = 6); for example, ‘I feel comfortable diagnosing or identifying a child as having autism’ (Helps et al., 1999).

(c) not applicable to current UK conceptualisation (*n* = 5); for instance, ‘What is Asperger syndrome?’ (Shah, 2001; Asperger syndrome is no longer a diagnostic label used within current diagnostic manuals).

(d) overly technical or not assessing lay knowledge (*n* = 2); for example, ‘On the basis of scientific research, which of the following biochemical, neurological or metabolic mechanisms are more likely to be associated with autism?’ (Shah, 2001).

(e) generalised or ambiguous claims that would be difficult to evidence as true for most or all cases (*n* = 13), for instance, ‘Children with autism need extra help to learn’ (Harrison et al., 2017a, 2017b) or ‘Children with autism can grow up to go to college and marry’ (Gillespie-Lynch et al., 2015).

(f) religious in nature (*n* = 2), for example, ‘Autism is caused by God or a supreme being’ (Harrison et al., 2017a, 2017b).

Item-level disagreements were discussed with a third co-author until consensus was reached. 56 items were removed, leaving a pool of items (*n* = 39). Language was rephrased within some items to render it up-to-date and/or less stigmatising, for example, avoiding the term ‘disorder’ and instead using ‘condition’ or just ‘autism’ and using identity-first (‘autistic people’) rather than person-first language (‘people with autism’) in accordance with community preferences (Bottema-Beutel et al., 2021; Kenny et al., 2015).

##### Expert Focus Group to Assess and Generate Items

Five autism experts (bringing a range of lived and professional expertise, including two who were diagnosed as autistic themselves; see Table 1 for details) were recruited to participate in a focus group to further assess the appropriateness of the pool of questionnaire items (*n* = 39). Participants received out-of-pocket expenses and refreshments. Additionally, experts who contributed outside of a paid role were compensated with vouchers (£50). Experts were asked to generate additional items and discuss ‘correct’ item responses. Decision-making on items to include and ‘correct’ answers was arrived at by consensus. Where consensus could not be established, the item was not included.. This resulted in the removal of seven items and addition of two items (see Fig. 2).

**Table 1:** Focus group participant expertise

	Expertise
Expert 1	Highly Specialist Speech and Language Therapist with extensive experience of working with families and autistic children in the UK and internationally; autism researcher
Expert 2	Autism trainer, consultant and researcher; personal and family experience of autism; runs national organization for autistic people
Expert 3	Personal and family experience of autism; autism trainer and researcher; sits on local and national committees relevant to autism
Expert 4	Experienced autism researcher; clinical and educational experience of autism; family experience of autism
Expert 5	Consultant Child and Adolescent Psychiatrist with expertise in autism; family experience of autism

##### Expert Consensus on Clarity and Correct Responses

To further check the clarity of items and establish consensus on correct responses, the resulting pool of 34 items (26 true/false and 8 multiple choice) was compiled into an online questionnaire using SelectSurvey software. The questionnaire instructions requested that respondents state if an item was not clear and then to select the correct answer. Individuals aged over 18 years and living in the UK with personal, professional or academic expertise in autism were recruited through social media, snowballing, and autism research networks (both formalised networks and informal networks, i.e., individuals sharing the survey with people within their personal networks related to autism research). 56 experts within the autism field completed the questionnaire. Participants received no compensation for participation. Participants were included in the sample as ‘experts’ if they had two or more of the following: qualification relevant to autism at degree level or higher (self-defined); work experience of two years or more with significant contact with autistic people (self-defined); personal diagnosis of autism; diagnosis of autism in a close family member or close friend. Items which did not achieve greater than 80% consensus on the ‘correct’ answer were removed (*n* = 5). Where ≥ 20% responses were rated as ‘not clear’, the item was either reworded or removed (*n* = 5). In addition, to simplify the questionnaire design and scoring, eight multiple-choice questions (*n* = 8) were converted into true/false questions (*n* = 12). This process resulted in an item pool of 38 true/false items.

##### Administration of Items with Lay Sample

The pool of 38 items was then administered to a lay sample (a sample of people within the community irrespective of their expertise in autism). 221 participants were recruited via online purposive opportunity sampling (general [non-autism-related] social media networks, such as Facebook groups established to bring together people within a specific UK geography). Social media posts invited people with no particular interest in or connection to autism to complete a questionnaire designed to assess knowledge of autism to help us see how well it is pitched for people who are not specialists in autism. Interested individuals then clicked a link through to the participant information sheet, consent form, affiliation questions, and draft questionnaire items. Participants were asked the same questions regarding any affiliation to autism as used in the ‘expert’ online questionnaire; details are provided in Table 2. Participants received no compensation for participation.

**Table 2:** Initial item pool lay sample participants’ relationship to autism (N = 221); numbers exceed N as some respondents selected multiple categories

Expertise/affiliation	N
Personal diagnosis of autism	7
Close family member or friend who is autistic	71
Worked in a job with significant contact with autistic people	27
Qualification relating to autism (degree or above)	48
No expertise/affiliation to autism	109

Descriptive data for each item were computed. There was a high level of correct responding with 18/38 items (47%) showing ceiling effects, being answered correctly by ≥ 90% respondents. 197 participants (89%) correctly responded to over 80% of items (31 out of a total 38; M = 33.33; SD = 2.76). Due to the high level of correct responding, we deemed that this provisional pool of items was not performing well for this lay sample. Accordingly, we did not carry out any further analyses and, instead, took measures to develop a pool containing more difficult questionnaire items.

##### Measures to Develop More Difficult Items

Four steps were taken in order to produce a pool of more difficult questionnaire items:

1. The 18 items with  ≥ 90% correct response rate were removed (for instance, “most children with autism lose acquired speech”)
2. Multiple-choice items were reinstated as they are less prone to correct guessing. This included items with a single correct response (signalled to the respondent by “choose one”) AND those with multiple correct responses (signalled to the respondent by “check all that apply”). Items (*n* = 6) were combined or extended to convert into multiple choice questions (*n* = 4; for example, “ASD is a mental health condition: True/False” AND “ASD is a neurodevelopmental disability: True/False” were combined into: “Autism is (choose all that apply)”. Some items did not lend themselves to conversion into multiple-choice and these were maintained in a true/false format.
3. Scoring was altered to reduce the risk of guessing artificially inflating total scores. For each question, a point was awarded for each *correct* response, and one point was awarded each *non-selected incorrect* answer. Each item also had the response option of “not sure” added to further deter participants from guessing answers. See the section below and Online Resource 1 for more information regarding scoring.
4. The autism experts from the original focus group (Table 2) were re-contacted and invited to generate ideas for additional questionnaire items to assess a more advanced understanding of autism. The group discussed potential items and decided on those to be added to the draft questionnaire. This new pool of items included both multiple choice (*n* = 9; for instance, “Well-known psychological theories of autism include [check all that apply: cognitive disinhibition / double empathy problem / theory of mind / functional apathy / reduced central coherence]” and true/false (*n* = 1; “Pica refers to eating or mouthing non-edible items”) formats.

This process generated a revised pool of questionnaire items with 15 true/false questions and 13 multiple choice items.

##### Expert Consensus on Clarity and Correct Responses (Revised Pool)

Experts within the autism field were recruited through autism research networks, social media and snowballing and invited to complete this new pool of items to assess clarity and establish consensus on correct answers. Expert status was assessed in the same way as for the initial pool of items (see above). 35 experts completed the item pool. They were all over 18 years of age and lived in the UK. 9 (26%) were autistic; 20 (57%) had an autistic close family member or close friend; 34 (97%) had worked for over two years in a job with significant contact with autistic people; 18 (51%) had a qualification related to autism at degree level or higher (with 10 (29%) with a PhD related to autism). Items remained in the questionnaire if experts met consensus of 70% or above for correct answers; this threshold was reduced from the 80% level applied in the first phase due to the need to include more difficult items (on which it would inevitably be harder to achieve consensus). Following this process, no entire questions were removed (all 28 questions remained), but five individual item responses were removed from multiple-choice questions due to not meeting the 70% consensus threshold.

##### Administration of Items with Lay Sample (Revised Pool)

The new pool of items was then compiled as an online questionnaire using SelectSurvey and a lay sample of participants was recruited by purposive opportunity sampling, repeating the procedure outlined in the section on ‘*Piloting of items with lay sample’* above. The lay sample consisted of 201 participants. Participants were again asked about their affiliation to autism. Please see Table 3 for summary of participant autism affiliations. Participants also self-rated on their autism knowledge on 0 (‘I have never heard of autism’) to 10 (‘I consider myself a world-leading expert on autism’) scale with a mean rating being 4.78 (SD = 1.81; range 1–9).

**Table 3:** Revised pool lay participants’ relationship to autism (N = 201); numbers exceed N as some respondents selected multiple categories

Expertise/affiliation	N
Personal diagnosis of autism	7
Parent or guardian of autistic child	31
Close family member or friend who is autistic	66
Worked in a job with significant contact with autistic people	63
Qualification relating to autism (degree or above)	52
No expertise/affiliation to autism	79

#### Part 2: Psychometric Analysis

##### Scoring

The 28 questions were first recoded into dichotomous items with each item corresponding to a specific response option for a given question. For every correctly selected option and every unselected ‘incorrect’ option, a score of 1 was given. For instance, for Question 1 “Autism is”, for which response options were (a) a neurodevelopmental condition (correct), (b) a learning disability (incorrect), (c) a mental health condition (incorrect), d) a neurodegenerative condition (incorrect), and e) not sure, a respondent can achieve a score between 0 and 4. When a respondent selects option (a) only, they are awarded the maximum total score of 4; a score of 1 for choosing the correct option (a), and an additional score of 1 for each non-selected incorrect option (b–d). If one of the incorrect response items (b–d) had been selected by the respondent, no mark would be awarded for that item.

A score of 0 was awarded for the entire question if the “not sure” response was selected. This results in five separate dichotomous items (select: 1, not selected: 0 for the correct options and the reverse for the incorrect ones) for Question 1, each corresponding to one of the response options. The derived dichotomous items were used in an item response theory model. The dichotomous items were also summed to create polytomous items, which were used in factor analysis with mixed data. To illustrate, in the case of Question 1, a correctly completed question had a score of 4 as a polytomous item.

##### Item Response Theory

The dichotomous items of KAQ were used in a two-parameter logistic model (2PL-IRT; Baker, 2001) to estimate the probability of a correct response to an item depending on one’s level of autism knowledge, denoted as theta (θ; Kamata & Bauer, 2008), and item characteristics, (also known as parameters, severity, discrimination ability, and information). The key parameters under item response theory (IRT) are difficulty and discrimination. The difficulty parameter (intercept), denoted as *b*, indicates the location of an item on the autism knowledge trait, where the probability of a correct response is 50%. Lower values of the difficulty parameter correspond to “easier” questions, and as such correct responses can be obtained from individuals at lower levels of autism knowledge. The discrimination parameter (slope), denoted as *a*, is analogous to factor loadings and describes how well an item can differentiate between those with different levels of autism knowledge. Higher values of the *a* parameter indicate greater ability of an item to discriminate between individuals with different levels of the autism knowledge and are reflected by steeper slope of the item characteristic curve (ICC). The reliability or measurement precision of an item to measure the underlying autism knowledge is characterised in IRT by the concept of information. Greater information corresponds to greater measurement precision, and it can be illustrated using the information function curve, which shows how the reliability of each item can vary throughout different levels of autism knowledge. The item information curve is the highest at the location of the difficulty parameter, since more discriminating ability provides more information.

The 2PL-IRT model was used to guide item selection with the aim of retaining items that are evenly distributed across a range of locations on the autism knowledge scale. Such a scale would allow for meaningful interpretations in group differences and change over time (Nguyen et al., 2014). The items were identified as problematic if they had low discrimination ability as indicated by low values of the *a* parameter.

There is little consensus in psychometric literature on the requirements of sample size for the estimation of IRT parameters (Edelen & Reeve, 2007). The requirements not only depend on test length but also on model complexity with more complex models requiring a larger sample size with current estimations for 2-PL models ranging from 250 to 500 participants (Drasgow, 1989; Harwell & Janosky, 1991; Stone, 1992). Due to the number of items in the scale (60 dichotomous items) and the number of participants in this study (N = 201), sample size requirements for the 2PL-IRT model were not met. To accommodate this, the 2PL-IRT model was run separately for correct options (for example for Question 1 it would be “A neurodevelopmental condition” item) and for distractors (“A learning disability”, “A mental health condition”, “A neurodegenerative condition”).

##### Dimensionality of KAQ-UK

Exploratory factor analysis (EFA) for mixed data was conducted on full lay sample (N = 201) using weighted least squares mean and variance adjusted (WLSMV; Muthen, 1984) in MPlus (Muthén & Muthén, 1998), which can accommodate a combination of categorical and continuous dependent variables in the model. The number of factors to be retained was guided by the number of eigenvalues above 1 (Guttman-Kaiser criterion; Guttman, 1954; Kaiser, 1960), and by comparing the number of sample eigenvalues that are larger than the mean of eigenvalues generated from 100 randomly simulated correlation matrices of both tetrachoric and polychoric correlations (parallel analysis; Horn, 1965). These results were visualised using Cattell’s (1966) scree plot. The factor selection process was coupled with evaluation of model fit while also taking into account the parsimony and interpretability of the solution (Vitoratou et al., 2023). A number of goodness of fit indices were used to evaluate model fit: the relative chi-square (relative χ^2^ values below 2 or 3 suggest reasonable fit when coupled with other fit indices that are in acceptable ranges; Hoelter, 1983, Hu & Bentler, 1999), Root Mean Square Error of Approximation (RMSEA values of below 0.05 indicate close fit; Steiger, 1990, Browne & Cudek, 1993), Comparative Fit Index (CFI values greater than 0.95 suggest close fit; Bentler, 1990; Hu & Bentler, 1999), the Tucker-Lewis Index (TLI values greater than 0.95 demonstrate close fit; Hu & Bentler, 1999; Tucker & Lewis, 1973), and standardized root mean residual (SRMR values that are below 0.5 indicate close fit; Hu & Bentler, 1999; Kline, 2016). Items that did not have strong loadings (< 0.4) on the main factor were considered problematic and to be omitted.

Eight to ten responses per item are considered sufficient for generalised linear latent variables models (Kyriazos, 2018). Omitting items based on 2PL-IRT investigations resulted in 18 polytomous items (which equals to 47 items in dichotomised form), which totals to sample size requirements of at least 144 participants (376 for dichotomised items). The number of participants in this study allowed for exploratory factor analysis on polytomous items but was not sufficient for additional confirmatory methods explorations.

##### Internal Consistency Reliability

Internal consistency of the scale was evaluated on polytomous items using Cronbach’s alpha (α; Cronbach, 1951) and McDonald’s Omega (ω; McDonald, 1999). Guidelines for alpha and omega values recommend the values of > 0.70 as indicative of satisfactory internal consistency (Nunnally & Bernstein, 1994). The homogeneity of the items was further assessed using corrected item-total correlations (ITC; values below 0.2 and above 0.8 indicate an item to be redundant) and alpha/omega if item deleted (AID/OID).

##### Differences in Score Based on Affiliation to Autism

Participants indicated their affiliation to autism and were allocated to five distinct groups. To address overlaps in affiliations, a hierarchy was created which prioritised lived experience: (1) personal diagnosis of autism; (2) parent/guardian of autistic child(ren) or adult(s); (3) close friend or family member who is autistic; (4) a qualification relevant to autism at degree level or above; (5) a job with significant contact with autistic people. Participants with no affiliation to autism were pooled into a separate group. The differences in total scores between autism-affiliation groups were evaluated using one-way ANOVA with hoc-post tests. The analysis was conducted in MPlus (Muthén & Muthén, 1998), STATA (StataCorp., 2021), R version 4.3.1 (R Core Team, 2023) and SPSS (IBM Corp, 2021). Parallel analysis was conducted using the R package *psych* (Revelle, 2023).

## Results

### Item Response Theory: Preliminary Investigations

The first step in the analysis was to run 2-PL-IRT on correct options to examine the probability of a correct response to an item. All items and item responses can be found in Online Resource 2. The estimated difficulty (*b*) and discrimination (*a*) parameters are presented in Online Resource 3, and the corresponding item characteristic curves and item information functions are depicted in Online Resource 4. The analysis showed 6 problematic items with respect to low discrimination ability which were omitted from further investigations. Item 7 (“A person’s facial features can help you identify whether or not they are autistic: *False*”) was both the least discriminating and the least difficult item. Item 15 (“Unusual reactions to how things smell, taste, look, feel, or sound means a person must have autism: *False*”) was the second least difficult item with low ability to differentiate between those with different levels of autism knowledge. The further least discriminating item with below average difficulty was Item 2 (“Autism is a brain based condition: *True*”). With respect to the problematic items that had above average difficulty, Item 10 (“In identical twins, where one has autism, the chance of the other twin having autism is: *77–98%*”) was the most difficult item with very low discriminating ability. The further least discriminating items with above average difficulty were Items 9_2 (“Known causes of autism include: *New changes or mutations in genes*”) and 12 (“Short sightedness happens commonly alongside autism: *False*”). All 6 items had low information at all ranges of autism knowledge.

Two more items, 16 (“Aggression is not a defining feature of autism: *True*”) and 23 (“Most autistic people need to know what to expect more than people who are not autistic: *True*”), had below average difficulty and relatively low discrimination ability and information at below average level of autism knowledge, and were thus removed to balance out the number of easier items to more difficult items.

Item 14 (“All autistic people have a skill in which they particularly excel: *False*”) and item 22 (“The percentage of UK autistic adults in full-time paid employment is around: *16%*”) were below and above average performing items respectively in terms of difficulty with relatively low discriminating ability. Further discussion amongst the authorship team highlighted that anecdotal feedback suggested that item 14 could be ambiguous and prone to misinterpretation by participants (for example, “skill” could be interpreted as day-to-day individual strengths rather than the intended interpretation of savant skills). Item 22 was considered too specific and to be potentially easily invalidated through future research. Therefore, these two items were further omitted.

With respect to distractors, Item 9_4 (“Known causes of autism include: *Vaccinations in early childhood*”) was the most problematic item due to being the least difficult, having no discriminating ability and providing no information. This item has been already identified as problematic with 2-PL-IRT on correct options and removed. Three more items (I8_2, I11_2, I1_1) have been recognised as potentially problematic due to relatively low discriminating ability but were retained in factor analysis for further explorations. Please see Online Resource 3 for all 2-PL parameters for distractors.

### Factor Analysis

Eighteen polytomous items underwent dimensionality investigations. The number of eigenvalues of the covariance matrix that were above 1 (5.22, 1.63, 1.59, 1.36, 1.10) suggested up to five factors according to Kaiser-Guttman criterion. Nonetheless, the close fit to the data was achieved with one-factor solution (relative χ^2^ = 1.30, RMSEA [90% CI] = 0.039 [0.020, 0.054], CFI = 0.93, TLI = 0.92, SRMR = 0.102). Four items (I08, I13, I25, I26) did not load strongly (< 0.4) on their main factor, and each item was omitted from the model one by one. The goodness of fit indices of the resulting solution showed close fit to the data except for SRMR, which nevertheless indicated acceptable fit (relative χ^2^ = 1.20, RMSEA [90% CI] = 0.032 [0.000, 0.053], CFI = 0.97, TLI = 0.97, SRMR = 0.097). All items had loadings of above 0.4 on the main factor. Exploring solutions with increasing number of factors resulted in more items not loading meaningfully on any factor and some items cross-loading on other factors. The one-factor solution was thus accepted (see Table 4 for loadings) and the final set of items underwent further item response theory investigations.

**Table 4:** EFA loadings for the polytomous items sorted by decreasing loadings (N = 201)

Item	Label	Knowledge of autism
I21	Which of the following features of language are sometimes found in autism	0.759*
I04	Autism is more frequently diagnosed in males than females	0.656*
I18	When autistic people try and hide their autistic features this is known as	0.607*
I06	One of the people who first described autism in the twentieth century was called	0.580*
I17	Autistic people do not show affection, even to close family members	0.580*
I03	Other names that have been used for types of autism are	0.547*
I19	Many autistic people are interested in making friends	0.526*
I24	Commonly used strategies to support understanding in autism include	0.524*
I11	Well known psychological theories relating to autism include	0.523*
I01	Autism is	0.511*
I28	Common adjustments for autistic children in school include	0.505*
I27	Medication has been proven to improve autism	0.452*
I20	Pica refers to eating or mouthing non-edible items	0.450*
I05	Autism affects around 1 in 3000 people	0.413*

^*^Loading significant at *p* < 0.001

### Item Response Theory: Final Items

**Fig. 3:**
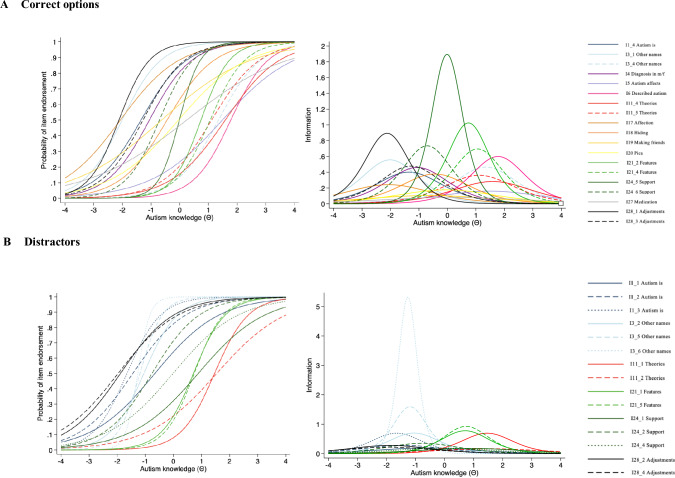
Item characteristic curves (ICC; on the left) and item information curves (IIF; on the right) for correct options (**A**) and distractors (**B**) for final set of items

2-PL-IRT was run on correct options for the final set of items identified through factor analysis (please see Table 5 for difficulty (*b*) and discrimination (*a*) parameters, and Fig. 3 for item characteristic curves and item information functions).

**Table 5:** 2-PL model parameters for correct options and distractors separately for final set of dichotomised items sorted by decreasing discrimination (a) values (N = 201)

Item	Label	Difficulty (*b*)	SE (*b*)	*p* value (*b*)	Discrimination (*a*)	SE (*a*)	*p* value (*a*)
*Correct options*							
I24_5	Commonly used strategies to support understanding in autism include: *Social Stories*	‒ 0.01	0.10	0.89	2.75	0.61	0.00
I21_2	Which of the following features of language are sometimes found in autism: *Echolalia*	0.74	0.14	0.00	2.02	0.41	0.00
I28_1	Common adjustments for autistic children in school include: *A quiet space*	‒ 2.12	0.38	0.00	1.89	0.58	0.00
I24_6	Commonly used strategies to support understanding in autism include: *Visual timetables*	‒ 0.71	0.15	0.00	1.71	0.36	0.00
I21_4	Which of the following features of language are sometimes found in autism: *Stereotyped language*	1.06	0.18	0.00	1.67	0.35	0.00
I6	One of the people who first described autism in the twentieth century was called: *Leo Kanner*	1.78	0.30	0.00	1.55	0.37	0.00
I3_1	Other names that have been used for types of autism are: *Asperger Syndrome*	‒ 2.01	0.39	0.00	1.49	0.43	0.00
I28_3	Common adjustments for autistic children in school include: *More explicit instructions*	‒ 1.28	0.24	0.00	1.38	0.32	0.00
I3_4	Other names that have been used for types of autism are: *Pervasive Developmental Disorder*	1.35	0.25	0.00	1.36	0.30	0.00
I4	Autism is more frequently diagnosed in males than females: *True*	‒ 1.06	0.21	0.00	1.36	0.30	0.00
I1_4	Autism is: *A neurodevelopmental condition*	‒ 1.35	0.27	0.00	1.27	0.31	0.00
I18	When autistic people try and hide their autistic features this is known as: *Masking*	‒ 0.44	0.16	0.01	1.23	0.26	0.00
I11_5	Well known psychological theories relating to autism include: *Theory of Mind*	1.11	0.23	0.00	1.20	0.26	0.00
I11_4	Well known psychological theories relating to autism include: *Reduced central coherence*	1.58	0.34	0.00	1.06	0.26	0.00
I17	Autistic people do not show affection, even to close family members: *False*	‒ 2.07	0.50	0.00	0.98	0.29	0.00
I20	Pica refers to eating or mouthing non-edible items: *True*	‒ 0.05	0.19	0.81	0.84	0.20	0.00
I5	Autism affects around 1 in 3000 people: *False*	1.48	0.39	0.00	0.80	0.21	0.00
I19	Many autistic people are interested in making friends: *True*	‒ 0.63	0.29	0.03	0.65	0.19	0.00
I27	Medication has been proven to improve autism: *False*	0.24	0.27	0.38	0.57	0.18	0.00
*Distractors*							
I3_6	Other names that have been used for types of autism are: *Williams Syndrome*	‒ 1.27	0.17	0.00	4.61	2.38	0.05
I3_5	Other names that have been used for types of autism are*: Tourette’s Syndrome*	‒ 1.19	0.18	0.00	2.53	0.76	0.00
I21_5	Which of the following features of language are sometimes found in autism: *Verb inversion*	0.74	0.15	0.00	1.93	0.47	0.00
I21_1	Which of the following features of language are sometimes found in autism: *Duality*	0.71	0.15	0.00	1.76	0.39	0.00
I3_2	Other names that have been used for types of autism are: *Dyspraxia*	‒ 1.05	0.19	0.00	1.67	0.38	0.00
I11_1	Well known psychological theories relating to autism include: *Cognitive disinhibition*	1.46	0.26	0.00	1.67	0.44	0.00
I1_3	Autism is: *A neurodegenerative condition*	‒ 1.65	0.28	0.00	1.67	0.42	0.00
I24_2	Commonly used strategies to support understanding in autism include: *Lip reading*	‒ 0.76	0.20	0.00	1.19	0.29	0.00
I1_2	Autism is: *A mental health condition*	‒ 1.44	0.32	0.00	1.07	0.27	0.00
I28_2	Common adjustments for autistic children in school include: *Bright and busy visual displays*	‒ 1.88	0.43	0.00	1.06	0.29	0.00
I28_4	Common adjustments for autistic children in school include: *More unstructured group work*	‒ 1.97	0.48	0.00	0.94	0.27	0.00
I1_1	Autism is: *A learning disability*	‒ 0.66	0.23	0.00	0.88	0.22	0.00
I24_4	Commonly used strategies to support understanding in autism include: *Number ladders*	‒ 0.01	0.19	0.95	0.87	0.24	0.00
I24_1	Commonly used strategies to support understanding in autism include: *Auditory scheduling*	0.88	0.28	0.00	0.85	0.23	0.00
I11_2	Well known psychological theories relating to autism include: *Functional apathy*	1.54	0.42	0.00	0.81	0.24	0.00

The least and most discriminating items were Item 27 (“Medication has been proven to improve autism: *False*”) and Item 24_5 (“Commonly used strategies to support understanding in autism include: *Social Stories*”), respectively. This indicates that Item 24_5 relates to larger, and Item 27 to smaller, differences in autism knowledge between those who endorse these items and who do not. With respect to difficulty, the location estimates of the items ranged from -2.12 to 1.78, showing wide variation in item location. Item 28_1 (“Common adjustments for autistic children in school include: *A quiet space*”) was identified as the least difficult and Item 6 (“One of the people who first described autism in the twentieth century was called: *Leo Kanner*”) as the most difficult one. Items were evenly distributed across a range of locations on the autism knowledge scale. Specifically, 6 items (I28_1, I17, I3_1, I1_4, I28_3, I4) were endorsed at lower levels of autism knowledge (Θ =  − 2 to ‒1), 7 items (I24_6, I19, I18, I20, I24_5, I27, I21_2) had average difficulty (Θ =  − 1 to 1), and 6 items (I21_4, I11_5, I3_4, I5, I11_4, I6) targeted higher trait levels of autism knowledge (Θ = 1 to 2), thus all items working together to represent a continuum of the trait. The information function curves were derived for each item, which indicated that Item 28_1 (“Common adjustments for autistic children in school include: *A quiet space*”) provided the most information for those with lower levels of autism knowledge, Item 24_5 (“Commonly used strategies to support understanding in autism include: *Social Stories*”) was most informative for people of average knowledge, and Item 6 (“One of the people who first described autism in the twentieth century was called: *Leo Kanner*”) provided the most information for high scorers.

In regard to distractors, the item with best discrimination ability was I3_6 (“Other names that have been used for types of autism are: *Williams Syndrome*”), and item 11_2 (“Well known psychological theories relating to autism include: *Functional apathy*”) was the least discriminating. The difficulty parameter ranged from − 1.97 to 1.54, with the least difficult item being Item 28_4 (“Common adjustments for autistic children in school include: *More unstructured group work*”), while the most difficult one being Item 11_2 (“Well known psychological theories relating to autism include: *Functional apathy*”). The information function curves for each item showed Item 3_6 (“Other names that have been used for types of autism are: *Williams Syndrome*”) to be the most informative for those at lower levels of the trait, and Item 21_5 (“Which of the following features of language are sometimes found in autism: *Verb inversion*”) had the greatest measurement precision for people with average to high levels of autism knowledge.

### Internal Consistency Reliability

Cronbach’s alpha and McDonald’s omega indicated satisfactory internal consistency, 0.714 and 0.733, respectively. No problematic items were identified with respect to corrected item-total correlations (0.263–0.506), and alpha (0.671–0.709) and omega (0.685–0.727) if item deleted.

### Differences in Score Based on Affiliation to Autism

There was a statistically significant difference between affiliation groups on the total score of KAQ-UK (*F*(5,195) = 6.534, *p* < 0.001). A post-hoc test (Tukey’s HSD) determined that parents of autistic people scored significantly higher than participants with no affiliation and those with autistic family member or a close friend. There were no further significant differences in total scores between the affiliation groups. Please see Table 6 for descriptive statistics and post-hoc tests.

**Table 6:** Categorised affiliation group differences in total scores on the KAQ-UK with one-way ANOVA post hoc tests.

Affiliation	N	Mean (SD)	95% CI for the mean	Autism diagnosis	Parent/carer of autistic person	Autistic family member/close friend	Qualification (degree or above) relating to autism	Job working with autistic people	No affiliation
Autism diagnosis	7	19 (7.39)	12.16–25.84	–					
Parent/carer of autistic person	29	23.38 (5.31)	21.36–25.40	0.462	–				
Autistic family member/close friend	47	18.68 (6.07)	16.90–20.46	1.00	**0.008**	–			
Qualification (degree or above) relating to autism	12	21.33 (5.85)	17.61–25.05	0.957	0.905	0.71	–		
Job working with autistic people	27	19.89 (5.66)	17.65–22.13	0.999	0.211	0.953	0.979	–	
No affiliation	79	16.66 (5.57)	15.41–17.90	0.906	** < .001**	0.399	0.096	0.123	–
Total	201	18.90 (6.13)	18.04–19.75						

Bolded values signify statistically significant differences between groups, *p* < 0.05.

### Associations Between Actual and Perceived Autism Knowledge

There was a moderate positive significant correlation between self-perceived autism knowledge, and total score on the KAQ-UK (*r* = 0.39, *p* < 0.001). A Pearson’s r correlation test revealed that the higher participants perceived their knowledge of autism to be, the higher their overall total score on the KAQ-UK.

## Discussion

This study aimed to develop and provide initial validation of a questionnaire of autism knowledge suitable to the current UK context. After a detailed item development process and initial psychometric investigations, we provide preliminary support for the validity of a 14-item questionnaire called the Knowledge of Autism Questionnaire-UK (KAQ-UK, see Online Resource 1 for the questionnaire and scoring guidelines). This questionnaire can be used to assess between-group differences in autism knowledge or to evaluate the impact of specific education, awareness-raising, or training activities on improving autism knowledge over time. Further evaluation and validation of its measurement properties are required.

### Strengths of the KAQ-UK

The new questionnaire has a number of strengths. First, it is brief (14 items) and therefore acceptable to be administered within a wider assessment battery without being too burdensome. Second, the item pool was generated and evaluated through an iterative development process with involvement from a range of experts with both personal and professional experience in autism. This has resulted in a questionnaire that holds face and content validity and is up-to-date and acceptable to a range of stakeholders in both its content and language. The questionnaire reflects contemporary understanding of the diversity of the autism spectrum and is focused on UK knowledge and practice, which can be different to US understanding and practice, and one of the main reasons that previous questionnaires (e.g., Harrison et al., 2017a, 2017b; McClain et al., 2019) were deemed unsuitable.

Third, we have paid close attention to two inter-related issues: (1) that the questionnaire is pitched at the right level of difficulty, and (2) that items can be evidenced as (almost) universally true or false. The initial stages of our development process highlighted that there was a better general understanding of autism within the lay validation sample than we had anticipated and therefore the first draft questionnaire showed ceiling effects. Consequently, we took measures to increase the difficulty of questionnaire items and reduce correct guessing. This included adding multiple choice questions and a ‘not sure’ response option. The heterogeneous nature of autism can render it difficult to isolate ‘true facts’ which hold across diverse presentations and experiences, and which can be universally evidenced as true or false. This presents challenges to questionnaire item generation. Many general statements of fact are difficult to evidence as true in all individual situations (e.g., autistic people can learn to drive). Loosely worded items (e.g., some autistic people can learn to drive) are easier to evidence as true or false but are prone to correct guessing and can therefore artificially inflate scores. All these factors taken together present challenges in developing questionnaire items that differentiate higher levels of knowledge (e.g., McClain et al., 2019; Stronach et al., 2019). To address these challenges, we carried out careful consensus with autism experts, to be confident in our decisions about correctness and incorrectness. In addition, we included KAQ-UK questionnaire items which are more ‘technical’ (e.g., the true/false item ‘Pica refers to eating or mouthing non-edible items’). To assess the KAQ-UK’s ability to differentiate between levels of knowledge, the 2PL-IRT analysis suggested that the 14 items on the final KAQ-UK were evenly distributed across low, average, and high trait levels of autism knowledge. These more technical items are therefore important to discriminate those with the highest levels of knowledge.

A further strength of the questionnaire is its thorough initial psychometric assessment. This is of particular significance given that previous research has shown that 57% of autism knowledge assessments have weak or no psychometric support (Harrison et al., 2017a, 2017b). The current study assessed the psychometric properties of the KAQ-UK in a number of ways. First, a 2PL-IRT model was employed to examine the difficulty, discrimination, and reliability of each item separately. Six items were removed due to lack of discrimination value and low information, suggesting low ability of the items to differentiate between people at different levels of knowledge and poor measurement precision for all ranges of autism knowledge. Two further items were removed due to low difficulty, discrimination, and information. As a result, the number of easier items to difficult became more balanced, thus reducing the risk of ceiling effects, with which autism knowledge assessments are commonly affected (for instance, Kuhn & Carter, 2006). Second, the dimensionality investigations revealed a one-factor model of the questionnaire that represents a single construct of autism knowledge. At this stage of analysis, four items were identified as not meaningful indicators of the knowledge of autism and were removed from the questionnaire. Further psychometric analysis showed satisfactory reliability estimated for the scale, indicating interitem consistency within the measure.

To assess discriminative validity of the tool, we asked the lay validation sample to indicate their affiliation to autism across several categories. This provided preliminary support discriminative validity. The scores of parents/carers of autistic people were significantly higher than those with no affiliation to autism, and those with an autistic close friend or family member. As we would expect parent/carers of autistic people to be more knowledgeable about autism than those with no or a looser affiliation to autism, this finding is reassuring. However, we did not observe significant group differences between scores obtained by people with no affiliation to autism, and those who worked in jobs with significant contact with autistic people, or had qualifications related to autism. The current study did not collect information regarding type of job or qualification, nor were the category descriptions operationalised for participants. As a result, poor group ascertainment may have affected the analysis of affiliation group differences of autism knowledge. Further exploration of discriminative validity is needed in future studies with more accurate categorization of individuals based on their autism-related qualifications to solidify these findings. Furthermore, the current study was unable to assess between group differences in scores obtained by autistic people. As only 7 participants within the lay validation sample reported that they were autistic themselves we were unable to assess between group differences in scores, due to low statistical power.

Furthermore, we also assessed if self-perceived autism knowledge was related to actual knowledge. Our results showed that overall scores on the KAQ-UK are positively related to self-perceived knowledge for the lay validation sample as a whole. We therefore believe this could act as a potential proxy for concurrent, convergent validity when no other similar measures are available.

### Limitations of the KAQ-UK

This study comes with limitations, primarily attributed to a limited sample size that did not allow for confirmatory factor analysis to be performed to evaluate the support of the factor structure derived from EFA. Additionally, the assessment of criterion-related validity was lacking. There is no ‘gold standard’ tool in this area and we decided to develop a questionnaire precisely because there were no other up-to-date UK-focussed questionnaires available. As a result, we cannot make claims regarding how the KAQ-UK’s compares to similar autism knowledge measures. Another limitation is that we have limited demographic information, such as gender, age and socio-economic class, of our lay validation sample, plus we relied upon social media and snowballing recruitment methods which can result in sampling bias. These factors make it difficult to make statements about representativeness and to evaluate if the measure and its results are fully applicable to the general population. Further validation of this new questionnaire is required before we can make confident statements about its performance within the general population.

Furthermore, as prompted by an anonymous reviewer of this work, the authors acknowledge that the term “I don’t know” can be read as a non-response option, therefore we suggest in future research and use of this scale this be replaced with the term “that is not within my knowledge”.

Further research is required to assess the appropriateness of this questionnaire for targeted groups and to evaluate its sensitivity to change over time and to detect important changes that result from awareness-raising campaigns or interventions. The inclusion of items which discriminate different levels of knowledge, as shown by item response theory, suggests that there may be scope to detect improvement over time. The KAQ-UK is currently being used in a large randomised controlled trial to evaluate the change in parent autism knowledge following a new post-diagnostic psychoeducation programme (REACH-ASD trial; Leadbitter et al., 2022). This will allow examination of the performance of the KAQ-UK with a more targeted group (parents and carers of children recently diagnosed with autism), its ability to detect change over time and the evaluation of whether the unidimensionality of the measure is supported in a new sample using confirmatory factor analysis. Anecdotal feedback from within this trial suggests that many parents/carers found the questionnaire acceptable and enjoyed completing a ‘quiz’ about their autism knowledge.

## Conclusion

In conclusion, the KAQ-UK is a brief 14-item assessment of UK-centred autism knowledge that has face validity and satisfactory psychometric properties. The questionnaire shows promise in being sensitive to change over time, although further validation is required. The KAQ-UK can be used across a variety of settings with a range of respondents to assess knowledge of autism. The questionnaire and scoring guidelines are freely available in Online Resource 1.

## Supplementary Information

Below is the link to the electronic supplementary material.

Supplementary file1 (DOCX 42 KB)

Supplementary file2 (DOCX 21 KB)

Supplementary file3 (DOCX 51 KB)

Supplementary file4 (DOCX 737 KB)
