# Correction: The Knowledge of Autism Questionnaire-UK: Development and Initial Psychometric Evaluation

**DOI:** 10.1007/s10803-024-06580-3

**Published:** 2024-10-10

**Authors:** Sophie Langhorne, Nora Uglik-Marucha, Charlotte Broadhurst, Elena Lieven, Amelia Pearson, Silia Vitoratou, Kathy Leadbitter

**Affiliations:** 1 Division of Psychology and Mental Health, University of Manchester, Oxford Road, Manchester, M13 9PL UK; 2 Psychometrics & Measurement Lab, Biostatistics and Health Informatics Department, King’s College London, London, SE5 8AF UK; 3 Department of Psychology, Communication and Human Neuroscience, University of Manchester, Oxford Road, Manchester, M13 9PL UK; 4 Division of Nursing, Midwifery & Social Work, University of Manchester, Oxford Road, Manchester, M13 9PL UK

**Correction to: Journal of Autism and Developmental Disorders**

10.1007/s10803-024-06332-3

In the Supplementary file 1 for this article, the value of total score ‘34’ should have read ‘38’.

The original article has been corrected.

