# Supplementary material for: The Knowledge of Autism Questionnaire-UK: Development and Initial Psychometric Evaluation
Source: J Autism Dev Disord. 2024 May 2;55(7):2436–51. doi: 10.1007/s10803-024-06332-3 (PMC12167355; doi:10.1007/s10803-024-06332-3)
Supplement: Supplementary file 1 — Supplementary file1 (DOCX 42 KB) [file 10803_2024_6332_MOESM1_ESM.docx]

**Online Resource 1: The Knowledge of Autism Questionnaire – UK: Questionnaire and Scoring Guidelines**

**The Knowledge of Autism Questionnaire - UK (KAQ-UK)**

Please answer each of the questions about autism below. If you don’t think you know the answer, please just select “not sure”.

**Autism is: (check ALL that apply)**

| A mental health condition | ☐ |
| --- | --- |
| A learning disability | ☐ |
| A neurodevelopmental condition | ☐ |
| A neurodegenerative condition | ☐ |
| Not sure | ☐ |

**Other names that have been** **used for types of autism are: (check ALL that apply)**

Asperger Syndrome ☐

Tourette’s syndrome ☐

Pervasive Developmental Disorder ☐

Dyspraxia ☐

Williams Syndrome ☐

Not sure ☐

**Autism is more frequently diagnosed in males than females:**

| True | ☐ |
| --- | --- |
| False | ☐ |
| Not sure | ☐ |

**Autism affects around 1 in 3000 people:**

| True | ☐ |
| --- | --- |
| False | ☐ |
| Not sure | ☐ |

**One of the first people who described autism in the 20^th^ Century was called: (choose one)**

Henry Fitzroyd ☐

Luke McKenzie ☐

Johann Cooper ☐

Leo Kanner ☐

Not sure ☐

**Well known psychological theories relating to autism include: (check ALL that apply)**

Cognitive disinhibition ☐

Theory of mind ☐

Functional apathy ☐

Reduced central coherence ☐

Not sure ☐

**Autistic people do not show affection, even to close family members:**

True ☐

False ☐

Not sure ☐

**When autistic people try and hide their autistic features this is known as: (choose one)**

| Fronting | ☐ |
| --- | --- |
| Masking | ☐ |
| Veiling | ☐ |
| Screening | ☐ |
| Not sure | ☐ |
|  |  |

**Many autistic people are interested in making friends:**

True ☐

False ☐

Not sure ☐

**Pica refers to eating or mouthing non-edible items:**

True ☐

False ☐

Not sure ☐

**Which of the following features of language are sometimes found in autism: (check ALL that apply)**

Stereotyped language ☐

Echolalia ☐

Duality ☐

Verb inversion ☐

Not sure ☐

**Commonly used strategies to support understanding in autism include: (check ALL that apply)**

| Auditory scheduling | ☐ |
| --- | --- |
| Visual timetable | ☐ |
| Number ladders | ☐ |
| Social stories | ☐ |
| Lip reading | ☐ |
| Not sure | ☐ |

**Medication has been proven to improve autism:**

| True | ☐ |
| --- | --- |
| False | ☐ |
| Not sure | ☐ |

**Common adjustments for autistic children in school include: (check ALL that apply)**

Bright and busy visual displays ☐

A quiet space ☐

More explicit instructions ☐

More unstructured group work ☐

Not sure ☐

**End of questionnaire**

**The Knowledge of Autism Questionnaire- UK (KAQ-UK) Scoring Guidelines**

The KAQ-UK is scored using both the right and wrong answers selected by participants. Questions which have a “choose one response” or are “true/ false” questions have a maximum score of 1. Questions of “check ALL that apply” have a maximum score based on the number of items available (e.g., a “check ALL” question with 6 potential answers has a maximum score of 6). Points are awarded to every item a participant selects correctly, as well as for every item a participant rightly deems as incorrect. Therefore, for every correctly selected answer, and every correct UNSELECTED wrong answer, give a score of 1. Please see the example below.

Each question also includes a “not sure” response. If a participant answers “not sure” as the sole answer to a question, 0 points are given. If a participant selects “not sure” as well as other item responses, they are not penalised or marked down for including “not sure” as an answer; treat this as though “not sure” is not selected.

**The colours of the United Kingdom flag are: (check ALL that apply)**

Red ☐

White ☐

Blue ☐

Green ☐

Yellow ☐

A maximum of 5 points can be awarded to this question.

Scenario 1: If the participant selects the three correct answers (red, white, and blue), and **ALSO** leaves the INCORRECT answers blank, they will get a total of 5 points (3 correct and 2 have correctly been deemed incorrect).

Complex scoring:

Scenario 2: If the participant chose the responses: red, white, and green they would have 2 correct answers (red and white) and 1 incorrect answer (green). As a result, the participant would lose a point for choosing the incorrect answer. The participant will also have deemed “blue” to be incorrect, therefore losing another mark. However, as the participant did not select “yellow” and therefore rightly deemed this response incorrect, they will receive another mark for correctly leaving the incorrect answer. As a result, the participant will receive a score of 3 for this question. See the table below for further information.

|  | **Total**  **Correctly selected** | **Total**  **Correctly**  **NOT**  **selected** | **Total**  **Incorrectly Selected** | **Total**  **Incorrectly**  **NOT**  **Selected** | **Total Correct** | **Total Incorrect** | **(TOTAL SCORE)** |
| --- | --- | --- | --- | --- | --- | --- | --- |
| Scenario 1 | 3  Red,  White, Blue | 2  Yellow,  Green | 0 | 0 | 5 | 0 | 5 |
| Scenario 2 | 2  Red, white | 1 Yellow | 1 Green | 1  Blue | 3 | 2 | 3 |

**See the next page for a table of correct and incorrect responses**

**KAQ-UK Answer Sheet**

| Question | Correct Answers | Incorrect Answers | Total Score available |
| --- | --- | --- | --- |
| 1  Autism is: (check ALL that apply) | A neurodevelopmental condition | A learning disability    A mental health condition    A neurodegenerative condition    Not Sure | 4 |
| 2  Other names that have been used for types of autism are: (check  ALL that apply) | Asperger Syndrome    Pervasive Developmental Disorder | Tourette’s Syndrome    Dyspraxia    Williams Syndrome    Not Sure | 5 |
| 3  Autism is more frequently diagnosed in males than females: | True | False    Not Sure | 1 (True/false question) |
| 4  Autism affects around 1 in 3000 people: | False | True    Not Sure | 1 (True/false question) |
| 5  One of the first people who described autism in the 20th Century was called: (choose one) | Leo Kanner | Henry Fitzroyd    Luke McKenzie  Johann Cooper    Not Sure | 1 (Choose one response) |
| 6  Well known psychological theories relating to autism include: (check  ALL that apply) | Theory of Mind    Reduced Central Coherence | Cognitive disinhibition    Functional Apathy    Not Sure | 4 |
| 7  Autistic people do not show affection, even to close family members. | False | True Not Sure | 1( True/False Question) |
| 8  When autistic people try and hide their autistic features this is known as: (choose one) | Masking | Fronting    Veiling    Screening    Not Sure | 1 (choose  1question) |
| 9  Many autistic people are interested in making friends: | True | False    Not Sure | 1 (True/false question) |
| 10  Pica refers to eating or mouthing non edible items: | True | False    Not Sure | 1 (True/false question) |
| 11  Which of the following features of language are sometimes found in autism: (check ALL that apply) | Stereotyped Language  Echolalia | Verb Inversion  Duality  Not Sure | 4 |
| 12    Commonly used strategies to support understanding in autism include: (check ALL that apply) | Visual Timetable    Social Stories | Auditory Scheduling    Number Ladders    Lip Reading    Not Sure | 5 |
| 13    Medication has been proven to improve autism: | False | True  Not Sure | 1 (True/false question) |
| 14    Common adjustments for autistic children in school include (check ALL that apply) | A Quiet Space  More Explicit Instructions | Bright and Busy Visual Displays  More Unstructured Group Work Not Sure | 4 |
|  |  | Total score: | 34 |
