# Supplementary material for: The Knowledge of Autism Questionnaire-UK: Development and Initial Psychometric Evaluation
Source: J Autism Dev Disord. 2024 May 2;55(7):2436–51. doi: 10.1007/s10803-024-06332-3 (PMC12167355; doi:10.1007/s10803-024-06332-3)
Supplement: Supplementary file 2 — Supplementary file2 (DOCX 21 KB) [file 10803_2024_6332_MOESM2_ESM.docx]

Online Resource 2: All items included in the Item Response Theory Model. Please note these items are not representative of the final questionnaire. Please find the final version of the KAQ-UK in Online Resource 1.

| Item | Correct Item Response(s) | Incorrect Item Response(s) |
| --- | --- | --- |
| Autism is: | A neurodevelopmental condition | A learning disability, A mental health condition, A neurodegenerative condition |
| Autism is a brain based condition: | True | False |
| Other names that have been used for types of autism are: | Asperger Syndrome, Pervasive developmental Disorder | Dyspraxia, Tourette's Syndrome, Williams Syndrome |
| Autism is more frequently diagnosed in males than females: | True | False |
| Autism affects around 1 in 3000 people: | False | True |
| One of the people who first described autism in the 20th century was called: | Leo Kanner | Henry Fitzroyd, Luke McKenzie, Johann Cooper |
| A person's facial features can help you identify whether or not they have autism: | False | True |
| To be diagnosed with autism a person needs to have: | Restricted, repetitive patterns of behaviour or interests, Social communication difficulties | Hyperactivity, Language delay, visuo-spatial difficulties |
| Known causes of autism include: | Genes being passed down from parents to their children, new changes or mutations in genes | Vaccinations in early childhood |
| In identical twins, where one is autistic, the chance of the other twin being autistic is: | 77-98% | 0-16%, 17-46%, 47-76% |
| Well known psychological theories relating to autism include: | Theory of mind, Reduced Central Coherence: | Cognitive disinhibition, functional apathy |
| Short sightedness happens commonly alongside autism: | False | True |
| Coordination difficulties happen commonly alongside autism: | True | False |
| All autistic people have a skill in which they particularly excel: | False | True |
| Unusual reactions to how things smell, taste, look, feel, or sound means a person must have autism: | False | True |
| Aggression is not a defining feature of autism: | True | False |
| Autistic people do not show affection, even to close family members: | False | True |
| When autistic people try and hide their autistic features this is known as: | Masking | Fronting, Veiling, Screening |
| Many autistic people are interested in making friends: | True | False |
| Pica refers to eating or mouthing non-edible items: | True | False |
| Which of the following features of language are sometimes found in autism: | Echolalia, Stereotyped language | Duality, Verb Inversion |
| The percentage of UK autistic adults in full-time paid employment is around: | 16% | 2%, 34%, 51% |
| Most autistic people need to know what to expect more than people who are not autistic: | True | False |
| Commonly used strategies to support understanding in autism include: | Visual timetable, Social Stories | Auditory Scheduling, Number Ladders, Lip Reading |
| Commonly used sensory aids in autism include: | Ear defenders, weighted blanket | Reinforced Soles, Silk Gloves, Protective Helmet, Plug-in diffuser |
| Dietary modifications have been proven to improve the symptoms of autism: | False | True |
| Medication has been proven to improve autism: | False | True |
| Common adjustments for autistic children with autism in school include: | A quiet space, More explicit instructions | Bright and busy visual displays, More unstructured group work |
