# Supplementary material for: The Knowledge of Autism Questionnaire-UK: Development and Initial Psychometric Evaluation
Source: J Autism Dev Disord. 2024 May 2;55(7):2436–51. doi: 10.1007/s10803-024-06332-3 (PMC12167355; doi:10.1007/s10803-024-06332-3)
Supplement: Supplementary file 3 — Supplementary file3 (DOCX 51 KB) [file 10803_2024_6332_MOESM3_ESM.docx]

Online Resource 3: 2-PL model parameters for correct options and distractors for initial dichotomised items sorted by decreasing discrimination (a) values (N=201).

| **Item Label** | | | **Difficulty (*b*)** | | **SE (*b*)** | | **p value (*b*)** | | **Discrimination (*a*)** | | **SE (*a*)** | | **p value (*a*)** | | |
| --- | --- | --- | --- | --- | --- | --- | --- | --- | --- | --- | --- | --- | --- | --- | --- |
| *Correct options* | | |  | |  | |  | |  | |  | |  | | |
| I24_5 | Commonly used strategies to support understanding in autism include: *Social Stories* | -0.01 | | 0.11 | | 0.95 | | 2.35 | | 0.44 | | 0.00 | |  |  |
| I21_2 | Which of the following features of language are sometimes found in autism: *Echolalia* | 0.75 | | 0.14 | | 0.00 | | 2.01 | | 0.39 | | 0.00 | |  |  |
| I24_6 | Commonly used strategies to support understanding in autism include: *Visual timetables* | -0.71 | | 0.15 | | 0.00 | | 1.75 | | 0.35 | | 0.00 | |  |  |
| I28_1 | Common adjustments for autistic children in school include: *A quiet space* | -2.23 | | 0.42 | | 0.00 | | 1.71 | | 0.51 | | 0.00 | |  |  |
| I4 | Autism is more frequently diagnosed in males than females: *True* | -0.97 | | 0.18 | | 0.00 | | 1.60 | | 0.33 | | 0.00 | |  |  |
| I3_1 | Other names that have been used for types of autism are: *Asperger Syndrome* | -1.96 | | 0.35 | | 0.00 | | 1.57 | | 0.42 | | 0.00 | |  |  |
| I8_5 | To be diagnosed with autism a person needs to have: *Social communication difficulties* | -1.72 | | 0.30 | | 0.00 | | 1.56 | | 0.38 | | 0.00 | |  |  |
| I6 | One of the people who first described autism in the 20th century was called: *Leo Kanner* | 1.81 | | 0.31 | | 0.00 | | 1.50 | | 0.36 | | 0.00 | |  |  |
| I21_4 | Which of the following features of language are sometimes found in autism: *Stereotyped language* | 1.15 | | 0.21 | | 0.00 | | 1.45 | | 0.30 | | 0.00 | |  |  |
| I25_1 | Commonly used sensory aids in autism include: *Ear defenders* | -0.90 | | 0.18 | | 0.00 | | 1.41 | | 0.29 | | 0.00 | |  |  |
| I1_4 | Autism is: *A neurodevelopmental condition* | -1.28 | | 0.23 | | 0.00 | | 1.40 | | 0.31 | | 0.00 | |  |  |
| I3_4 | Other names that have been used for types of autism are: *Pervasive Developmental Disorder* | 1.36 | | 0.25 | | 0.00 | | 1.35 | | 0.29 | | 0.00 | |  |  |
| I28_3 | Common adjustments for autistic children in school include: *More explicit instructions* | -1.34 | | 0.25 | | 0.00 | | 1.29 | | 0.29 | | 0.00 | |  |  |
| I11_5 | Well known psychological theories relating to autism include: *Theory of Mind* | 1.10 | | 0.23 | | 0.00 | | 1.22 | | 0.26 | | 0.00 | |  |  |
| I18 | When autistic people try and hide their autistic features this is known as: *Masking* | -0.45 | | 0.16 | | 0.01 | | 1.19 | | 0.24 | | 0.00 | |  |  |
| I25_7 | Commonly used sensory aids in autism include: *Weighted blanket* | -0.43 | | 0.16 | | 0.01 | | 1.19 | | 0.24 | | 0.00 | |  |  |
| I11_4 | Well known psychological theories relating to autism include: *Reduced central coherence* | 1.64 | | 0.36 | | 0.00 | | 1.01 | | 0.25 | | 0.00 | |  |  |
| I17 | Autistic people do not show affection, even to close family members: *False* | | -2.05 | | 0.48 | | 0.00 | | 1.00 | | 0.28 | | 0.00 |  |  |
| I20 | Pica refers to eating or mouthing non-edible items: *True* | | -0.04 | | 0.19 | | 0.82 | | 0.85 | | 0.20 | | 0.00 |  |  |
| I22 | The percentage of UK autistic adults in full-time paid employment is around: *16%* | | 1.66 | | 0.42 | | 0.00 | | 0.81 | | 0.22 | | 0.00 |  |  |
| I5 | Autism affects around 1 in 3000 people: *False* | | 1.51 | | 0.39 | | 0.00 | | 0.79 | | 0.21 | | 0.00 |  |  |
| I19 | Many autistic people are interested in making friends: *True* | | -0.59 | | 0.26 | | 0.02 | | 0.70 | | 0.19 | | 0.00 |  |  |
| I13 | Coordination difficulties happen commonly alongside autism: *True* | | -0.22 | | 0.24 | | 0.36 | | 0.67 | | 0.18 | | 0.00 |  |  |
| I23 | Most autistic people need to know what to expect more than people who are not autistic: *True* | | -2.73 | | 0.89 | | 0.00 | | 0.66 | | 0.24 | | 0.01 |  |  |
| I14 | All autistic people have a skill in which they particularly excel*: False* | | -1.06 | | 0.36 | | 0.00 | | 0.65 | | 0.19 | | 0.00 |  |  |
| I16 | Aggression is not a defining feature of autism: *True* | | -2.19 | | 0.70 | | 0.00 | | 0.63 | | 0.21 | | 0.00 |  |  |
| I27 | Medication has been proven to improve autism: *False* | | 0.23 | | 0.26 | | 0.38 | | 0.59 | | 0.18 | | 0.00 |  |  |
| I9_1 | Known causes of autism include: *Genes being passed down from parents to their children* | | 0.17 | | 0.27 | | 0.54 | | 0.57 | | 0.17 | | 0.00 |  |  |
| I8_4 | To be diagnosed with autism a person needs to have: *Restricted, repetitive patterns of behaviour or interests* | | -2.23 | | 0.78 | | 0.00 | | 0.56 | | 0.20 | | 0.01 |  |  |
| I26 | Dietary modifications have been proven to improve the symptoms of autism: *False* | | 1.28 | | 0.48 | | 0.01 | | 0.54 | | 0.18 | | 0.00 |  |  |
| I2 | Autism is a brain based condition: *True* | | -3.17 | | 1.35 | | 0.02 | | 0.47 | | 0.21 | | 0.03 |  |  |
| I12 | Short sightedness happens commonly alongside autism: *False* | | 1.11 | | 0.51 | | 0.03 | | 0.45 | | 0.17 | | 0.01 |  |  |
| I9_2 | Known causes of autism include: *New changes or mutations in genes* | | 0.82 | | 0.50 | | 0.10 | | 0.39 | | 0.17 | | 0.02 |  |  |
| I10 | In identical twins, where one is autistic, the chance of the other twin being autism is*: 77-98%* | | 4.84 | | 2.80 | | 0.08 | | 0.37 | | 0.22 | | 0.09 |  |  |
| I15 | Unusual reactions to how things smell, taste, look, feel, or sound means a person must be autistic: *False* | | -4.68 | | 2.71 | | 0.09 | | 0.37 | | 0.22 | | 0.10 |  |  |
| I7 | A person's facial features can help you identify whether or not they are autistic: *False* | | -18.67 | | 41.59 | | 0.65 | | 0.10 | | 0.23 | | 0.65 |  |  |

*Distractors*

I24_2 Commonly used strategies to support understanding in autism include: *Lip reading* -0.57 0.13 0.00 2.08 0.49 0.00

| I24_4 | Commonly used strategies to support understanding in autism include: *Number ladders* | -0.01 | 0.12 | 0.93 | 1.91 | 0.43 | 0.00 |  |  |
| --- | --- | --- | --- | --- | --- | --- | --- | --- | --- |
| I24_1 | Commonly used strategies to support understanding in autism include: *Auditory scheduling* | 0.55 | 0.14 | 0.00 | 1.81 | 0.39 | 0.00 |  |  |
| I25_4 | Commonly used sensory aids in autism include: *Protective helmet* | -0.15 | 0.13 | 0.23 | 1.71 | 0.39 | 0.00 |  |  |
| I25_3 | Commonly used sensory aids in autism include: *Plug-in diffuser* | -0.67 | 0.16 | 0.00 | 1.68 | 0.45 | 0.00 |  |  |
| I25_5 | Commonly used sensory aids in autism include: *Reinforced soles* | -0.51 | 0.16 | 0.00 | 1.45 | 0.38 | 0.00 |  |  |
| I25_6 | Commonly used sensory aids in autism include: *Silk gloves* | -0.65 | 0.17 | 0.00 | 1.41 | 0.38 | 0.00 |  |  |
| I28_2 | Common adjustments for autistic children in school include: *Bright and busy visual displays* | -1.70 | 0.34 | 0.00 | 1.23 | 0.32 | 0.00 |  |  |
| I3_6 | Other names that have been used for types of autism are: *Williams Syndrome* | -2.18 | 0.50 | 0.00 | 1.18 | 0.36 | 0.00 |  |  |
| I28_4 | Common adjustments for autistic children in school include: *More unstructured group work* | -1.69 | 0.35 | 0.00 | 1.17 | 0.30 | 0.00 |  |  |
| I21_1 | Which of the following features of language are sometimes found in autism: *Duality* | 0.98 | 0.25 | 0.00 | 1.02 | 0.26 | 0.00 |  |  |
| I11_1 | Well known psychological theories relating to autism include: *Cognitive disinhibition* | 2.03 | 0.51 | 0.00 | 0.98 | 0.30 | 0.00 |  |  |
| I3_2 | Other names that have been used for types of autism are*: Dyspraxia* | -1.51 | 0.37 | 0.00 | 0.95 | 0.26 | 0.00 |  |  |
| I8_6 | To be diagnosed with autism a person needs to have: *Visuo-spatial difficulties* | -1.19 | 0.30 | 0.00 | 0.94 | 0.24 | 0.00 |  |  |
| I1_3 | Autism is: *A neurodegenerative condition* | -2.42 | 0.64 | 0.00 | 0.93 | 0.29 | 0.00 |  |  |
| I21_5 | Which of the following features of language are sometimes found in autism: *Verb inversion* | 1.17 | 0.31 | 0.00 | 0.90 | 0.24 | 0.00 |  |  |
| I3_5 | Other names that have been used for types of autism are: *Tourette's Syndrome* | -2.19 | 0.60 | 0.00 | 0.86 | 0.28 | 0.00 |  |  |
| I8_1 | To be diagnosed with autism a person needs to have: *Hyperactivity* | -1.22 | 0.34 | 0.00 | 0.82 | 0.22 | 0.00 |  |  |
| I1_2 | Autism is: *A mental health condition* | -2.11 | 0.66 | 0.00 | 0.66 | 0.22 | 0.00 |  |  |
| I1_1 | Autism is: *A learning disability* | -1.04 | 0.46 | 0.02 | 0.50 | 0.19 | 0.01 |  |  |
| I11_2 | Well known psychological theories relating to autism include: *Functional apathy* | 2.42 | 1.02 | 0.02 | 0.48 | 0.21 | 0.02 |  |  |
| I8_2 | To be diagnosed with autism a person needs to have: *Language delay* | -0.81 | 0.44 | 0.07 | 0.46 | 0.18 | 0.01 |  |  |
| I9_4 Known causes of autism include: *Vaccinations in early childhood* -16.83 100.98 0.87 0.03 0.16 0.87 | | | | | | | | | |
