# Supplementary material for: The Knowledge of Autism Questionnaire-UK: Development and Initial Psychometric Evaluation
Source: J Autism Dev Disord. 2024 May 2;55(7):2436–51. doi: 10.1007/s10803-024-06332-3 (PMC12167355; doi:10.1007/s10803-024-06332-3)

Online Resource 4: Item characteristic curves (ICC; on the left) and item information curves (IIF; on the right) for correct options (A) and distractors (B) for initial set of items


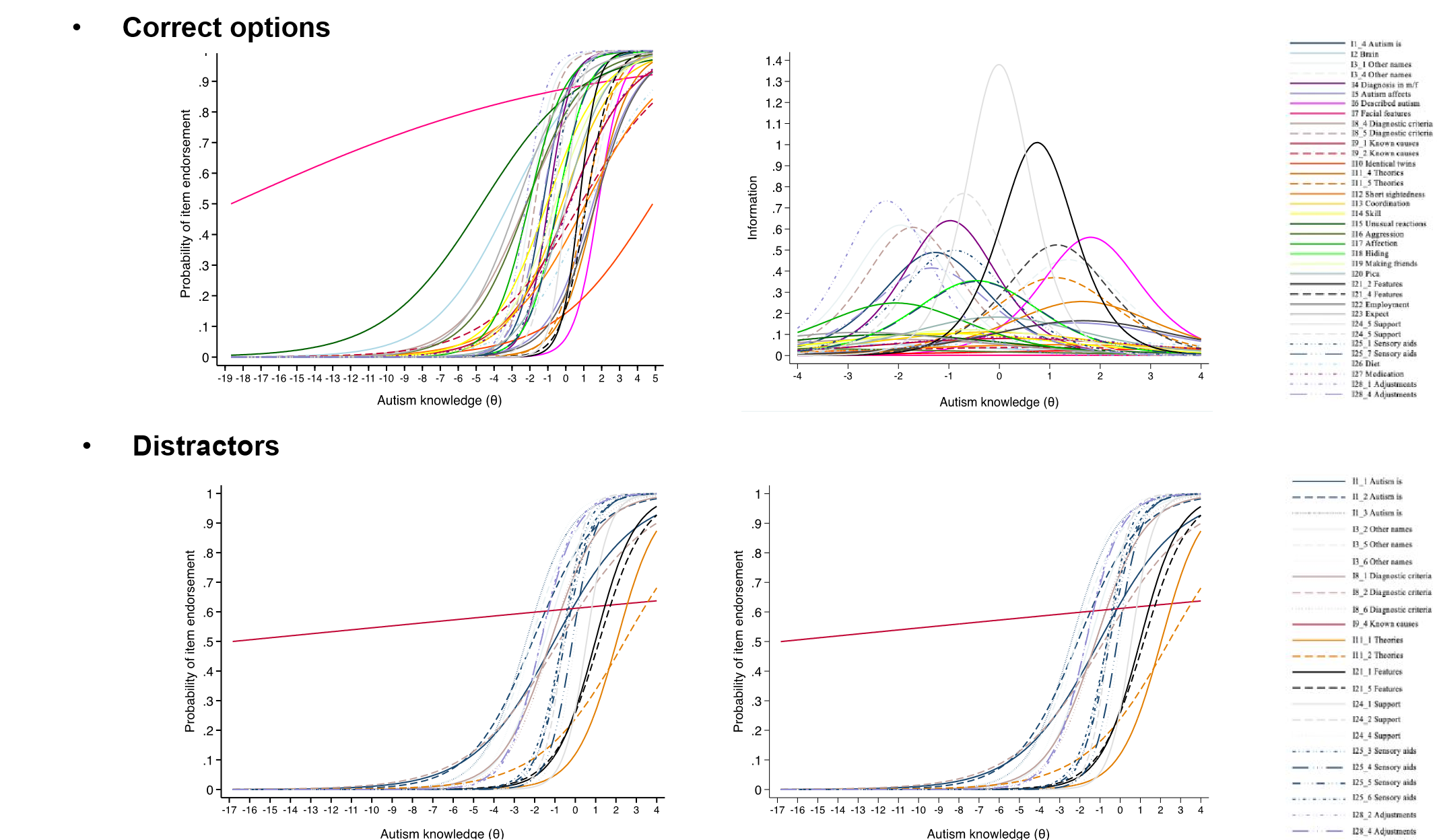

Supplement: Supplementary file 4 — Supplementary file4 (DOCX 737 KB) [file 10803_2024_6332_MOESM4_ESM.docx]
